# Perception of Local COVID-19 Transmission and Use of Preventive Behaviors Among Adults with Recent SARS-CoV-2 Infection — Illinois and Michigan, June 1–July 31, 2022

**DOI:** 10.15585/mmwr.mm7146a2

**Published:** 2022-11-18

**Authors:** Mark É. Czeisler, Rashon I. Lane, Robert C. Orellana, Kristen Lundeen, Kathryn Macomber, Jim Collins, Prerna Varma, Lauren A. Booker, Shantha M.W. Rajaratnam, Mark E. Howard, Charles A. Czeisler, Brendan Flannery, Matthew D. Weaver

**Affiliations:** ^1^Francis Weld Peabody Society, Harvard Medical School, Boston, Massachusetts; ^2^Turner Institute for Brain and Mental Health and School of Psychological Sciences, Monash University, Melbourne, Victoria, Australia; ^3^Institute for Breathing and Sleep, Austin Health, Heidelberg, Victoria, Australia; ^4^Division of Sleep and Circadian Disorders, Departments of Medicine and Neurology, Brigham and Women’s Hospital, Boston, Massachusetts; ^5^Division of Sleep Medicine, Harvard Medical School, Boston, Massachusetts; ^6^Sutter Health, Sacramento, California; ^7^Michigan Department of Health and Human Services; ^8^CDC Foundation COVID-19 Corps, Atlanta, Georgia; ^9^DuPage County Health Department, Wheaton, Illinois; ^10^La Trobe University, Bendigo, Victoria, Australia; ^11^University of Melbourne, Melbourne, Victoria, Australia; ^12^CDC COVID-19 Emergency Response Team.

During the early stages of the COVID-19 pandemic, use of preventive behaviors was associated with perceived risk for contracting SARS-CoV-2 infection (*1*,*2*). Over time, perceived risk has declined along with waning COVID-19–related media coverage (*3*,*4*). The extent to which communities continue to be aware of local COVID-19 transmission levels and are implementing recommended preventive behaviors is unknown. During June 1–July 31, 2022, health departments in DuPage County, Illinois and metropolitan Detroit, Michigan surveyed a combined total of 4,934 adults who had received a positive test result for SARS-CoV-2 during the preceding 3 weeks. The association between awareness of local COVID-19 transmission and use of preventive behaviors and practices was assessed, both in response to perceived local COVID-19 transmission levels and specifically during the 2 weeks preceding SARS-CoV-2 testing. Both areas had experienced sustained high COVID-19 transmission during the study interval as categorized by CDC COVID-19 transmission levels.* Overall, 702 (14%) respondents perceived local COVID-19 transmission levels as high, 987 (20%) as substantial, 1,902 (39%) as moderate, and 581 (12%) as low; 789 (16%) reported they did not know. Adjusting for geographic area, age, gender identity, and combined race and ethnicity, respondents who perceived local COVID-19 transmission levels as high were more likely to report having made behavioral changes because of the level of COVID-19 transmission in their area, including wearing a mask in public, limiting travel, and avoiding crowded places or events. Continued monitoring of public perceptions of local COVID-19 levels and developing a better understanding of their influence on the use of preventive behaviors can guide COVID-19 communication strategies and policy making during and beyond the pandemic.

During June 1–July 31, 2022, adults aged ≥18 years who had received positive SARS-CoV-2 test results within the preceding 3 weeks who were reported to six participating health departments^†^ were invited via SMS text messages to complete anonymous, English-language Internet-based questionnaires as part of the COVID-19 Outbreak Public Evaluation (COPE) Initiative.^§^ The number of surveys sent to eligible potential respondents during this interval is not known. Respondents self-reported demographic information and the number of COVID-19 vaccine doses they had received. Respondents also 1) characterized levels of local COVID-19 transmission when surveyed as high, substantial, moderate, low, or unknown^¶^; 2) classified their level of concern about new variants of SARS-CoV-2; 3) reported frequency of having used preventive behaviors, including wearing a mask in public (and mask type worn), limiting travel, and avoiding crowded places or events, during the 2 weeks preceding SARS-CoV-2 testing**; and 4) reported changes in these preventive behaviors in response to perceived levels of local COVID-19 transmission.^††^

This analysis reviewed survey responses from participating health departments with 1,000 or more respondents during the study interval, which included the metropolitan area of Detroit, Michigan (including Lapeer, Livingston, Macomb, Oakland, St. Clair, and Wayne counties) and DuPage County, Illinois. During June 1–July 31, 2022, a total of 5,575 persons from the Detroit metropolitan area, who had received a positive SARS-CoV-2 test result opened the survey, 4,274 (76.7%) of whom completed the survey; 3,934 (92.0%) of these respondents provided information for all of the variables included in this analysis (except for general health status) and were included in the analytic sample.^§§^ Also during this interval, 1,546 persons from DuPage County, Illinois who had received a positive SARS-CoV-2 test result opened the survey; 1,207 (78.1%) completed the survey and 1,000 (83.0%) of these respondents provided information for all of the variables included in this analysis and were added to the analytic sample. Pearson’s chi-square tests were used to compare perceived local COVID-19 transmission across demographic groups, by number of vaccine doses received, and respondents’ concern about new variants of SARS-CoV-2. To assess associations between perceived local COVID-19 transmission level and frequency of use of preventive behaviors during the 2 weeks before SARS-CoV-2 testing and changes in personal behaviors due to perceptions of local COVID-19 transmission, adjusted odds ratios (aORs) were estimated using multivariable logistic regression models^¶¶^ adjusted for geographic area, gender identity,*** age group, and combined race and ethnicity. Respondents provided consent electronically. Analyses were conducted using Python software (version 3.8.8; Python Software Foundation) and R software (version 4.2.0; R Foundation) using the R survey package (version 3.29). The Monash University Human Research Ethics Committee reviewed and approved the study. This activity was reviewed by CDC and conducted consistent with applicable federal law and CDC policy.^†††^

Respondents, all adults, included 3,934 residents of the Detroit metropolitan area and 1,000 residents of DuPage County, Illinois. A total of 4,670 (94.6%) surveys were completed within 7 days of associated positive SARS-CoV-2 test results; all surveys were completed within 3 weeks of the associated positive test result.

During May–July 2022 (i.e., the study interval and reference time frame of questions answered by respondents), the Detroit metropolitan area and DuPage County had continuously high levels of local COVID-19 transmission as categorized by publicly available CDC transmission levels.^§§§^ Among all respondents, 702 (14%) characterized local COVID-19 transmission when surveyed as high, 971 (20%) as substantial, 1,902 (39%) as moderate, 581 (12%) as low, and 778 (16%) did not know (Table). Perceived level of local COVID-19 transmission varied by county, gender identity, age group, race and ethnicity, education, employment status, number of COVID-19 vaccine doses received, self-reported general health status, and respondents’ level of concern about new variants of SARS-CoV-2. Respondents aged 30–59 years were more likely than those aged 18–29 years or ≥60 years to characterize local COVID-19 transmission as high. High perceived local COVID-19 transmission levels were also more common among adults with relatively higher education attainment, more concern about new SARS-CoV-2 variants, and receipt of more COVID-19 vaccine doses. Higher percentages of adults with a high school diploma or less, zero COVID-19 vaccine doses, and no expressed concern about new variants of SARS-CoV-2 indicated that they did not know the level of COVID-19 transmission in their local area.

**TABLE:** Perception of local COVID-19 transmission among adults with recent positive SARS-CoV-2 test results — Illinois and Michigan, June 1–July 31, 2022

Characteristic	Perception of local COVID-19 transmission when surveyed, no. (%)						p-value*
	All	Don’t know	Low	Moderate	Substantial	High	
**Overall**	**4,934 (100.0)**	**778 (15.8)**	**581 (11.8)**	**1,902 (38.5)**	**971 (19.7)**	**702 (14.2)**	**NA**
**Survey completion interval**							
Jun 1–15	**1,179 (23.9)**	152 (12.9)	91 (7.7)	408 (34.6)	270 (22.9)	258 (21.9)	<0.001
Jun 16–30	**1,067 (21.6)**	160 (15.0)	160 (15.0)	452 (42.4)	178 (16.7)	117 (11.0)	
Jul 1–15	**1,341 (27.2)**	242 (18.0)	173 (12.9)	534 (39.8)	240 (17.9)	152 (11.3)	
Jul 16–31	**1,347 (27.3)**	224 (16.6)	157 (11.7)	508 (37.7)	283 (21.0)	175 (13.0)	
**Residence^†^**							
**Detroit, Michigan, metropolitan area**	**3,934 (79.7)**	652 (16.6)	494 (12.6)	1,528 (38.8)	775 (19.7)	485 (12.3)	<0.001
Lapeer County	**33 (0.7)**	3 (9.1)	9 (27.3)	13 (39.4)	7 (21.2)	1 (3.0)	
Livingston County	**176 (3.6)**	34 (19.3)	27 (15.3)	79 (44.9)	22 (12.5)	14 (8.0)	
Macomb County	**761 (15.4)**	136 (17.9)	96 (12.6)	327 (43.0)	124 (16.3)	78 (10.2)	
Oakland County	**1,487 (30.1)**	226 (15.2)	169 (11.4)	585 (39.3)	332 (22.3)	175 (11.8)	
Saint Clair County	**103 (2.1)**	9 (8.7)	32 (31.1)	28 (27.2)	21 (20.4)	13 (12.6)	
Wayne County	**1,374 (27.8)**	244 (17.8)	161 (11.7)	496 (36.1)	269 (19.6)	204 (14.8)	
**DuPage County, Illinois**	**1,000 (20.3)**	126 (12.6)	87 (8.7)	374 (37.4)	196 (19.6)	217 (21.7)	
**Gender**							
Female	**3,194 (64.7)**	520 (16.3)	337 (10.6)	1,230 (38.5)	621 (19.4)	486 (15.2)	0.013
Male	**1,676 (34.0)**	245 (14.6)	237 (14.1)	652 (38.9)	339 (20.2)	203 (12.1)	
Other or unknown	**64 (1.3)**	13 (20.3)	7 (10.9)	20 (31.3)	11 (17.2)	13 (20.3)	
**Age group, yrs**							
18–29	**638 (12.9)**	127 (19.9)	54 (8.5)	258 (40.4)	136 (21.3)	63 (9.9)	<0.001
30–44	**1,393 (28.2)**	210 (15.1)	111 (8.0)	511 (36.7)	294 (21.1)	267 (19.2)	
45–59	**1,579 (32.0)**	237 (15.0)	201 (12.7)	604 (38.3)	290 (18.4)	247 (15.6)	
≥60	**1,323 (26.8)**	204 (15.4)	214 (16.2)	529 (40.0)	251 (19.0)	125 (9.4)	
**Race and ethnicity**							
Asian, non-Hispanic	**322 (6.5)**	57 (17.7)	45 (14.0)	131 (40.7)	56 (17.4)	33 (10.2)	<0.001
Black, non-Hispanic	**575 (11.7)**	137 (23.8)	64 (11.1)	192 (33.4)	111 (19.3)	71 (12.3)	
Hispanic or Latino, any race or races	**262 (5.3)**	49 (18.7)	25 (9.5)	98 (37.4)	48 (18.3)	42 (16.0)	
White, non-Hispanic	**3,693 (74.8)**	518 (14.0)	434 (11.8)	1,457 (39.5)	736 (19.9)	548 (14.8)	
Other race or races, non-Hispanic	**82 (1.7)**	17 (20.7)	13 (15.9)	24 (29.3)	20 (24.4)	8 (9.8)	
**Highest level of education**							
High school diploma or less	**437 (8.9)**	121 (27.7)	65 (14.9)	147 (33.6)	54 (12.4)	50 (11.4)	<0.001
College or some college	**2,905 (58.9)**	494 (17.0)	346 (11.9)	1,124 (38.7)	561 (19.3)	380 (13.1)	
More than bachelor's degree	**1,592 (32.3)**	163 (10.2)	170 (10.7)	631 (39.6)	356 (22.4)	272 (17.1)	
**Employment status**							
Employed	**3,796 (76.9)**	575 (15.1)	418 (11.0)	1,483 (39.1)	761 (20.0)	559 (14.7)	0.017
Not employed	**1,138 (23.1)**	203 (17.8)	163 (14.3)	419 (36.8)	210 (18.5)	143 (12.6)	
**No. of COVID-19 vaccine doses received^§^**							
0	**252 (5.1)**	68 (27.0)	40 (15.9)	90 (35.7)	26 (10.3)	28 (11.1)	<0.001
1	**75 (1.5)**	14 (18.7)	10 (13.3)	34 (45.3)	10 (13.3)	7 (9.3)	
2	**921 (18.7)**	190 (20.6)	121 (13.1)	349 (37.9)	146 (15.9)	115 (12.5)	
3	**2,865 (58.1)**	417 (14.6)	305 (10.6)	1,091 (38.1)	618 (21.6)	434 (15.1)	
4	**821 (16.6)**	89 (10.8)	105 (12.8)	338 (41.2)	171 (20.8)	118 (14.4)	
**Self-reported health status^¶^**							
Excellent	**962 (19.5)**	126 (13.1)	151 (15.7)	345 (35.9)	182 (18.9)	158 (16.4)	<0.001
Very good	**2,200 (44.7)**	317 (14.4)	277 (12.6)	863 (39.2)	445 (20.2)	298 (13.5)	
Good	**1,355 (27.5)**	243 (17.9)	126 (9.3)	546 (40.3)	269 (19.9)	171 (12.6)	
Fair	**356 (7.2)**	81 (22.8)	24 (6.7)	125 (35.1)	67 (18.8)	59 (16.6)	
Poor	**53 (1.1)**	10 (18.9)	2 (3.8)	19 (35.8)	8 (15.1)	14 (26.4)	
**Level of concern about new variants of SARS-CoV-2**							
Not at all concerned	**287 (5.8)**	85 (29.6)	68 (23.7)	82 (28.6)	29 (10.1)	23 (8.0)	<0.001
Somewhat unconcerned	**331 (6.7)**	40 (12.1)	57 (17.2)	132 (39.9)	60 (18.1)	42 (12.7)	
Neutral	**939 (19.0)**	161 (17.1)	135 (14.4)	406 (43.2)	153 (16.3)	84 (8.9)	
Somewhat concerned	**2,312 (46.9)**	309 (13.4)	244 (10.6)	946 (40.9)	482 (20.8)	331 (14.3)	
Very concerned	**1,065 (21.6)**	183 (17.2)	77 (7.2)	336 (31.5)	247 (23.2)	222 (20.8)	

**Abbreviation:** NA = not applicable.

* Pearson’s chi-square tests were used to estimate p-values for differences across groups. A Bonferroni adjustment (10) was applied to account for the number of comparisons.

^†^ During June 1–July 31, 2022, a total of 45,626 confirmed COVID-19 cases occurred in the metropolitan area of Detroit, Michigan, and 18,626 confirmed COVID-19 cases in DuPage County, Illinois.

^§^ Respondents answered the question, “How many COVID-19 vaccine doses have you received?”

^¶^ Percentages for this group are derived from among the 4,926 respondents who self-reported health status. Eight of the 4,934 (0.2%) respondents did not complete the question on self-reported health status.

Multivariable models revealed that perceived higher local COVID-19 transmission among respondents was associated with more frequent participation in preventive behaviors during the 2 weeks preceding SARS-CoV-2 testing (Figure 1). Compared with respondents who characterized COVID-19 transmission as low, those who perceived transmission levels as high were more likely to report having always or often worn masks in public settings (aOR = 3.0; 95% CI = 2.3–3.8), to have worn protective masks (aOR = 2.9; 95% CI = 2.2–3.7), limited travel (aOR = 1.7; 95% CI = 1.3–2.1), and avoided crowded places or events (aOR = 1.6; 95% CI = 1.3–2.0).

**FIGURE 1:**
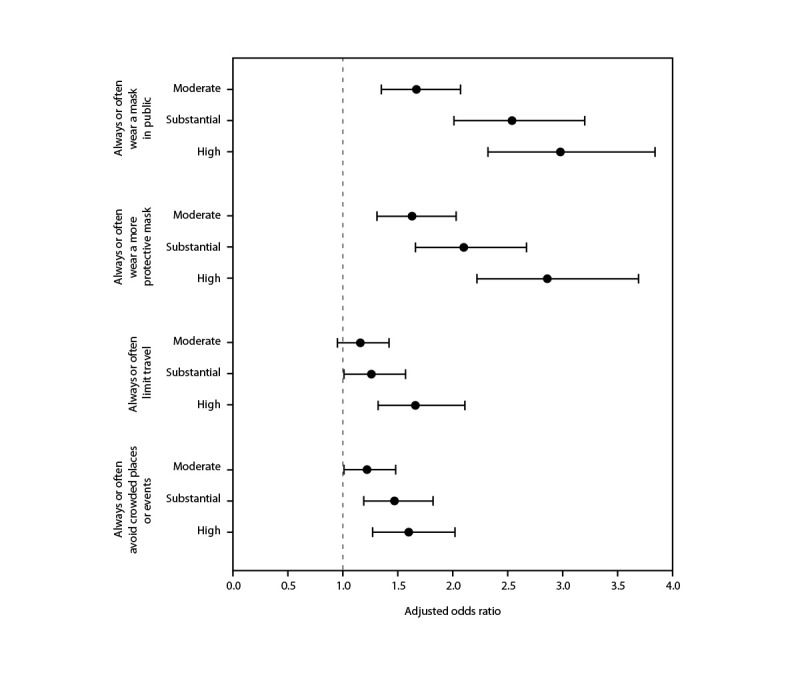
Adjusted odds ratios* of participation in preventive behaviors,^†^ by perceived level of local COVID-19 transmission^§¶^ among adults with recent positive SARS-CoV-2 test results — Illinois and Michigan, June 1–July 31, 2022 * With 95% CIs indicated by error bars Multivariable regression models are adjusted for geographic area, gender identity, age group, and combined race and ethnicity. ^†^ Self-reported preventive behaviors were ascertained with the lead question, “Generally speaking, during the two weeks before your most recent COVID-19 test, how often would you say you were doing each of the following to protect against COVID-19?” Response options were “Never,” “Rarely,” “Sometimes,” “Often,” and “Always.” Models estimated odds of having “Always” or “Often” versus “Rarely” or “Never” used preventive behaviors, omitting “Sometimes” given the imprecision of this answer. Among 4,934 respondents, the numbers of respondents in each model (i.e., excluding persons who reported “Sometimes” for the preventive behavior) were as follows: wearing a mask in public (3,646); choosing to wear a more protective mask (3,768); limiting travel (3,792); and avoiding crowded places or events (3,668). ^§^ Referent group = low transmission. ^¶^ The group of respondents who selected “I don’t know” for local COVID-19 transmission (778) is not included.

Compared with respondents who characterized local COVID-19 transmission as low, those who perceived local COVID-19 transmission as high were more likely to report changing their preventive behaviors in response to local transmission levels (aOR = 4.4; 95% CI = 3.2–5.0), substantial (aOR = 4.0; 95% CI = 3.2–5.0), or moderate (aOR = 2.1; 95% CI = 1.8–2.6) (Figure 2). Respondents who characterized local COVID-19 transmission as high were more likely than were those who characterized transmission as low to report having more frequently worn masks in public (aOR = 2.6; 95% CI = 1.7–4.1), chosen to wear a more protective mask (aOR = 1.7; 95% CI = 1.2–2.3), postponed or cancelled travel plans (aOR = 2.1; 95% CI = 1.4–3.1), and avoided crowded places or events (aOR = 2.0; 95% CI = 1.4–2.8).

**FIGURE 2:**
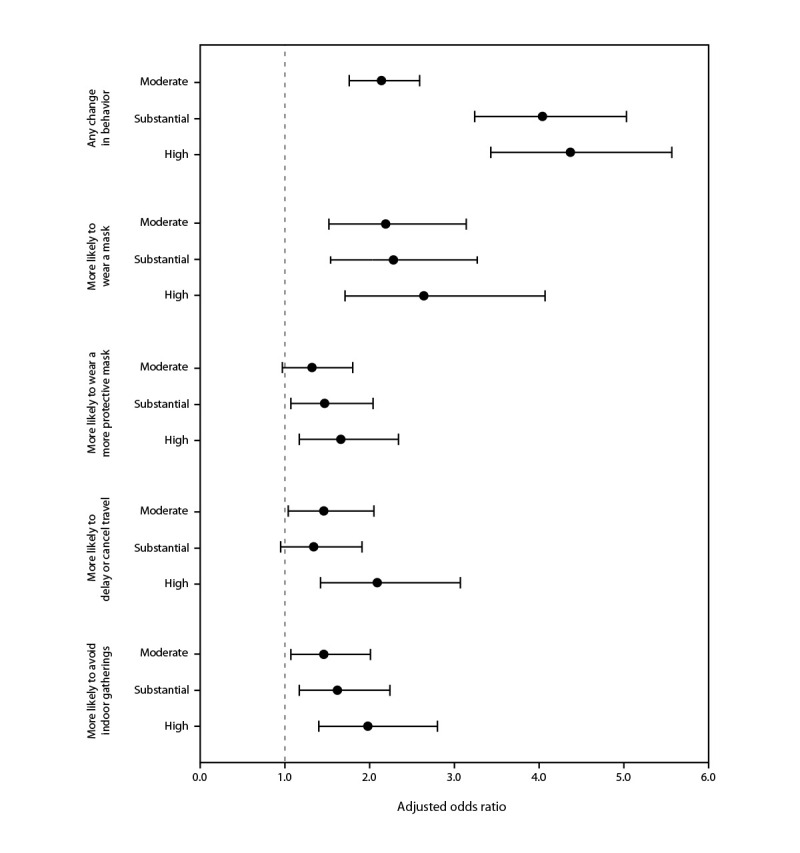
Adjusted odds ratios* for having changed use of preventive behaviors^†^ in response to perceived level of local COVID-19 transmission^§^^,^^¶^ among adults with recent positive SARS-CoV-2 test results — Illinois and Michigan, June 1–July 31, 2022 **Abbreviation:** Ref = referent group. * With 95% CIs indicated by error bars. Multivariable regression models are adjusted for geographic area, gender identity, age group, and combined race and ethnicity. ^†^ Respondents first answered “Yes” or “No” to the question, “Have you changed your behavior due to the level of COVID-19 transmission in your local area?” Respondents who answered “Yes” received the branching question, “In which of the following ways have you changed behavior?” for wearing a mask, choosing to wear a more protective mask, delaying or avoiding travel, or avoiding indoor gatherings with response options of “More likely,” “Unchanged,” “Less likely,” or “Not applicable.” Models estimated odds of any behavior change (versus no change) and higher likelihood (versus less likely or equally likely) of engaging in each preventive behavior, excluding persons who said they were not applicable. ^§^ Ref = low transmission. ^¶^ The group of respondents who selected “I don’t know” for local COVID-19 transmission (778) is not included.

## Discussion

In two geographic areas with sustained high 7-day average rates of confirmed COVID-19 transmission during May–July 2022, 50% of adults with recent SARS-CoV-2 infections surveyed during June–July 2022 described the level of COVID-19 transmission in their local area as low or moderate. Persons who perceived local COVID-19 transmission to be high when surveyed were most likely to report changing preventive behaviors in response to local COVID-19 transmission, including more frequently wearing a mask in public, limiting travel, and avoiding crowded events. Further assessment of public perceptions of local COVID-19 levels and their associations with preventive behaviors can help to clarify how communication of pandemic indicators and related policy decisions might influence behaviors.

Differences in perceived local COVID-19 transmission observed across demographic groups, number of vaccine doses received, and concern about new variants of SARS-CoV-2 highlight the effects of individual risk perception on use of preventive measures. Differences in perceived transmission levels among adults aged 30–59 years and those who were older or younger might reflect differential sources of COVID-19 information or COVID-19 risk perception (*6*). Perceived transmission level also varied with the number of COVID-19 vaccine doses received. Despite higher risk for severe COVID-19 without vaccine-induced protection, adults who had received fewer COVID-19 vaccine doses more commonly characterized COVID-19 transmission as low compared with adults who had received more COVID-19 vaccine doses. This finding might reflect a decreased likelihood to get vaccinated and to pay attention to COVID-19 transmission levels among people who were less concerned about COVID-19. In addition, even among persons who were very concerned about new variants of SARS-CoV-2, only one in five perceived local COVID-19 transmission to be high, which might be related to reduced media coverage of COVID-19 (*4*).

CDC does not recommend that members of the public use transmission levels alone to guide prevention measures. Rather, CDC developed COVID-19 Community Levels, which are measures of the impact of COVID-19 on a community in terms of hospitalizations and health care system strain, while accounting for transmission in the community. As such, calculation of COVID-19 Community Levels incorporates new COVID-19 hospital admissions and percentage of hospital beds occupied by patients with COVID-19, in addition to new COVID-19 cases in a community (*7*). Although not available at the time this survey was developed,^¶¶¶^ CDC recommends use of COVID-19 Community Levels data to guide messaging about community and individual preventive actions (*8*).

The findings in this report are subject to at least five limitations. First, questionnaires were completed by adults who had recently received a positive SARS-CoV-2 test result, which could have influenced their perceptions about local COVID-19 transmission levels. Relatedly, perceived local COVID-19 transmission levels when surveyed might have differed from perceived transmission levels during reference intervals for behaviors and practices, though transmission levels in both areas were sustainably high during the entire study interval and reference time frame (May–July 2022). Second, some respondents might have been aware of the CDC COVID-19 Community Level site and responded to survey questions accordingly, resulting in relatively lower reported perceived local COVID-19 transmission levels. Third, respondents might have overreported use of preventive behaviors because of social desirability (*9*), and this study did not assess whether reported behavioral changes occurred before or after respondents received a positive SARS-CoV-2 test result. Fourth, this nonrandom convenience sample is subject to selection bias related to COVID-19 test-seeking, and the survey sample does not represent all county residents who received a positive SARS-CoV-2 test result during the study interval. Finally, the number of persons who received survey invitations and were eligible to consent to participate is unknown, precluding a reliable response rate estimate.

This analysis found that a low percentage of surveyed U.S. adults perceived local COVID-19 transmission to be high despite sustained documented high transmission levels, and that those who perceived local transmission to be high were more likely to practice behaviors to protect themselves and others from COVID-19. Continued monitoring of public perceptions of local COVID-19 levels, and developing a better understanding of their influence on use of preventive behaviors, can guide COVID-19 communication strategies and policy making during and beyond the pandemic.

**Summary**

**What is already known about this topic?**

During June–July 2022, many U.S. counties experienced high COVID-19 transmission levels.

**What is added by this report?**

One half of adults surveyed during June–July 2022 who had recently received a positive SARS-CoV-2 test result in metropolitan Detroit, Michigan and DuPage County, Illinois perceived local COVID-19 transmission when surveyed to be low or moderate, despite documented sustained high transmission. Higher perceived local COVID-19 transmission was associated with more use of preventive behaviors, overall and in response to high local COVID-19 transmission.

**What are the implications for public health practice?**

Continued monitoring of public perceptions of local COVID-19 levels, and further understanding their impact on use of preventive behaviors, can guide pandemic-related communication strategies and policymaking.

## Footnotes

* CDC transmission levels are categorized as low, moderate, substantial, or high based on new COVID-19 case counts and the percentage of positive COVID-19 tests. CDC transmission levels, used for comparison with public perceptions, are available through the COVID Data Tracker. https://covid.cdc.gov/covid-data-tracker/#county-view?list_select_state=all_states&list_select_county=all_counties&data-type=Risk&null=Risk (Accessed November 8, 2022).

† Participating health departments included Clay County Health Department, Hayes, North Carolina; Public Health Madison and Dane County, Madison, Wisconsin; Denver Department of Public Health and Environment, Denver, Colorado; DuPage County Health Department, Wheaton, Illinois, Michigan Department of Health and Human Services, and New Mexico Department of Health.

§ The COPE Initiative surveys included in this analysis were designed for rapid administration to persons identified and recruited through county and state health departments. The COPE Initiative case-control surveys were established in February 2021. https://www.thecopeinitiative.org/

¶ Survey respondents were asked, “Which of the following would you use to describe the level of COVID-19 transmission in your local area?” with response options of low, moderate, substantial, high, and unknown.

** Participants were asked, “During the two weeks before your most recent COVID-19 test, how often would you say you were doing each of the following to protect against COVID-19?” Response options were “Never,” “Rarely,” “Sometimes,” “Often,” and “Always.” For this analysis, response options were collapsed into categories of never or rarely and often or always; responses of sometimes were excluded from analyses.

†† Survey respondents were asked about changes in personal preventive behavior with the question, “Have you changed your behavior due to the level of COVID-19 transmission in your local area?” Respondents who reported changes in behavior were asked whether they were more likely, less likely or just as likely to wear a mask, choose a more protective mask, delay or avoid travel, or avoid indoor gatherings. Responses of “not applicable” were excluded.

§§ The general health variable was added to the table after inclusion criteria had been established and the data locked on the final analytic sample; therefore, the table specifically indicates that eight of the 4,934 (0.2%) respondents in the final sample did not provide information on this variable.

¶¶ A subset of characteristics was included in multivariable regression models given inherent collinearity (e.g., between age and employment status or age and education attainment, or between concern about SARS-CoV-2 variants and COVID-19 vaccine status). Commonly assessed variables were included to guide potential tailoring of public health messaging about associations (e.g., by gender identity, age, or combined race and ethnicity).

*** To assess gender identity, respondents were asked, “What is your gender? (select one)” with response options of “male,” “female,” “trans, male/trans man,”” trans female/trans woman,” “genderqueer/gender nonconforming,” “different identity (please state),” and “prefer not to say.” For this analysis, gender identities were categorized as male, female, and other or unknown (including trans male/trans man, trans female/trans woman, genderqueer/gender nonconforming, different identity [please state], and prefer not to say).

††† 45 C.F.R. part 46, 21 C.F.R. part 56; 42 U.S.C. Sect. 241(d); 5 U.S.C. Sect. 552a; 44 U.S.C. Sect. 3501 et seq.

§§§ During the study period, the mean 7-day new COVID-19 case counts per 100,000 population for each county were as follows: DuPage County, Illinois = 257.6; Lapeer County, Michigan = 94.9; Livingston County, Michigan = 139.7; Macomb County, Michigan = 185.6; Oakland County, Michigan = 198.7; Saint Clair County, Michigan = 118.2; Wayne County, Michigan = 174.2. The mean 7-day percentage reported SARS-CoV-2 positive test results for each county were DuPage County, Illinois = 13.6%; Lapeer County, Michigan = 16.8%; Livingston County, Michigan = 14.6%; Macomb County, Michigan = 17.8%; Oakland County, Michigan = 16.8%; Saint Clair County, Michigan = 19.0%; and Wayne County, Michigan = 11.1%. https://covid.cdc.gov/covid-data-tracker/#county-view?list_select_state=all_states&data-type= (Accessed October 4, 2022).

¶¶¶ CDC Community Levels database was first available in March 2022. https://www.cdc.gov/coronavirus/2019-ncov/your-health/covid-by-county.html

## References

[1] Bruine de Bruin W, Bennett D. Relationships between initial COVID-19 risk perceptions and protective health behaviors: a national survey. Am J Prev Med 2020;59:157–67. 10.1016/j.amepre.2020.05.001 32576418 PMC7242956

[2] Czeisler MÉ, Garcia-Williams AG, Molinari NA, Demographic characteristics, experiences, and beliefs associated with hand hygiene among adults during the COVID-19 pandemic—United States, June 24–30, 2020. MMWR Morb Mortal Wkly Rep 2020;69:1485–91. 10.15585/mmwr.mm6941a3 33056951 PMC7561087

[3] Cipolletta S, Andreghetti GR, Mioni G. Risk perception towards COVID-19: a systematic review and qualitative synthesis. Int J Environ Res Public Health 2022;19:4649. 10.3390/ijerph19084649 35457521 PMC9028425

[4] Pearman O, Boykoff M, Osborne-Gowey J, COVID-19 media coverage decreasing despite deepening crisis. Lancet Planet Health 2021;5:e6–7. 10.1016/S2542-5196(20)30303-X 33421410

[5] Massetti GM, Jackson BR, Brooks JT, Summary of guidance for minimizing the impact of COVID-19 on individual persons, communities, and health care systems—United States, August 2022. MMWR Morb Mortal Wkly Rep 2022;71:1057–64. 10.15585/mmwr.mm7133e1 35980866 PMC9400529

[6] Ali SH, Foreman J, Tozan Y, Capasso A, Jones AM, DiClemente RJ. Trends and predictors of COVID-19 information sources and their relationship with knowledge and beliefs related to the pandemic: nationwide cross-sectional study. JMIR Public Health Surveill 2020;6:e21071. 10.2196/21071 32936775 PMC7546863

[7] CDC. COVID-19. COVID-19 by county. Atlanta, GA: US Department of Health and Human Services, CDC; 2022. Accessed August 8, 2022. https://www.cdc.gov/coronavirus/2019-ncov/your-health/covid-by-county.html

[8] Christie A, Brooks JT, Hicks LA, Sauber-Schatz EK, Yoder JS, Honein MA; CDC COVID-19 Response Team. Guidance for implementing COVID-19 prevention strategies in the context of varying community transmission levels and vaccination coverage. MMWR Morb Mortal Wkly Rep 2021;70:1044–7. 10.15585/mmwr.mm7030e2 34324480 PMC8323553

[9] Jakubowski A, Egger D, Nekesa C, Lowe L, Walker M, Miguel E. Self-reported vs directly observed face mask use in Kenya. JAMA Netw Open 2021;4:e2118830. 10.1001/jamanetworkopen.2021.18830 34328505 PMC8325070

